# Multiomics approach reveals the ubiquitination-specific processes hijacked by SARS-CoV-2

**DOI:** 10.1038/s41392-022-01156-y

**Published:** 2022-09-07

**Authors:** Gang Xu, Yezi Wu, Tongyang Xiao, Furong Qi, Lujie Fan, Shengyuan Zhang, Jian Zhou, Yanhua He, Xiang Gao, Hongxiang Zeng, Yunfei Li, Zheng Zhang

**Affiliations:** 1 Institute for Hepatology, National Clinical Research Center for Infectious Disease, Shenzhen Third People’s Hospital, The Second Affiliated Hospital, School of Medicine, Southern University of Science and Technology, 518112 Shenzhen, Guangdong Province China; 2 Guangzhou Laboratory, Guangzhou Medical University, Guangzhou, China; 3 Department of Microbiology, Second Military Medical University, Shanghai Key Laboratory of Medical Biodefense, 200433 Shanghai, China; 4 Guangdong Key laboratory for anti-infection Drug Quality Evaluation, 518112 Shenzhen, Guangdong Province China; 5 Shenzhen Research Center for Communicable Disease Diagnosis and Treatment of Chinese Academy of Medical Science, Shenzhen, Guangdong Province China

**Keywords:** Infectious diseases, Infectious diseases

## Abstract

The Coronavirus Disease 2019 (COVID-19) caused by Severe Acute Respiratory Syndrome Coronavirus 2 (SARS-CoV-2) is a global pandemic that seriously threatens health and socioeconomic development, but the existed antiviral drugs and vaccines still cannot yet halt the spread of the epidemic. Therefore, a comprehensive and profound understanding of the pathogenesis of SARS-CoV-2 is urgently needed to explore effective therapeutic targets. Here, we conducted a multiomics study of SARS-CoV-2-infected lung epithelial cells, including transcriptomic, proteomic, and ubiquitinomic. Multiomics analysis showed that SARS-CoV-2-infected lung epithelial cells activated strong innate immune response, including interferon and inflammatory responses. Ubiquitinomic further reveals the underlying mechanism of SARS-CoV-2 disrupting the host innate immune response. In addition, SARS-CoV-2 proteins were found to be ubiquitinated during infection despite the fact that SARS-CoV-2 itself didn’t code any E3 ligase, and that ubiquitination at three sites on the Spike protein could significantly enhance viral infection. Further screening of the E3 ubiquitin ligases and deubiquitinating enzymes (DUBs) library revealed four E3 ligases influencing SARS-CoV-2 infection, thus providing several new antiviral targets. This multiomics combined with high-throughput screening study reveals that SARS-CoV-2 not only modulates innate immunity, but also promotes viral infection, by hijacking ubiquitination-specific processes, highlighting potential antiviral and anti-inflammation targets.

## Introduction

The causative agent of Coronavirus Disease 2019 (COVID-19) pandemic, Severe Acute Respiratory Syndrome Coronavirus 2 (SARS-CoV-2) has infected hundreds of millions of people and killed millions, causing major health and economic crises.^1^ Currently, antiviral drugs have been springing up in laboratory and some are even entering clinical trials, but treatment options for COVID-19 are still limited, especially for severe cases. The emergency use of vaccines has made a substantial contribution to the epidemic prevention and control, but the constant emergence of SARS-CoV-2 variants greatly challenges the effectiveness of the vaccines. Therefore, an in-depth understanding of the pathogenesis of SARS-CoV-2 is urgently needed to identify more effective therapeutic targets.

SARS-CoV-2 infection causes a variety of symptoms and immunological perturbations have been shown to correlate with COVID-19 severity.^2–4^ However, the molecular mechanism of how SARS-CoV-2 infection directly disrupts host immunity remains unclear. Several omics studies have expanded our understanding of the pathophysiology of COVID-19.^5–8^ Metabolomic and proteomic analysis of patient specimens further clarified that an excessive immune response is the main driver of severe pneumonia.^9,10^ Proteomic analysis of SARS-CoV-2-infected cells revealed intracellular signaling pathways modulated by SARS-CoV-2 and provides some new antiviral targets.^8^ Modificationomics further revealed the changes in phosphorylation or ubiquitination of key proteins in these signaling pathways.^5–7^ However, these studies did not use lung epithelial cells but instead of Vero, Huh7 or cell lines artificially overexpressing ACE2, which fails to mimic the natural infection of SARS-CoV-2. Therefore, it is crucial to investigate the pathways that are relevant for SARS-CoV-2 pathogenicity in epithelial cells by unbiased omics approaches.

Ubiquitination is a common and important posttranslational modification (PTM) involved in almost all aspects of eukaryotic biology,^11^ requiring the sequential action of activating (E1), conjugating (E2) and ligating (E3) enzymes.^12^ Ubiquitination plays a complex and multi-faceted regulatory role in the process of viral infection, which can act on the signaling pathway of the host immune response or directly modify the viral proteins. For example, the ubiquitination of RIG-I^13^ and MAVS^14^ are important for interferon (IFN) production. The E3 ligase TRIM7 promotes enterovirus 2BC protein degradation to defend against virus infection.^15^ On the contrary, virus can also encode E3 ligases to mediate host protein ubiquitination for immune escape.^16^ A previous study showed that not only the host proteins but also the viral proteins were largely ubiquitinated during SARS-CoV-2 infection.^6^ However, the effect of viral proteins ubiquitination on virus pathogenicity has not been thoroughly investigated.

In this study, we first profiled the transcriptome, proteome and ubiquitinome of SARS-CoV-2-infected lung epithelial cells, and characterized the innate immune responses and underlying ubiquitination mechanisms. More importantly, our data identified a large number of ubiquitinated viral proteins and demonstrated that ubiquitination at three sites on the Spike protein affects viral infection. Subsequently, we identified four E3 ubiquitin ligases that affect viral infection by high-throughput screening. This study provides unbiased insights into the ubiquitination-specific processes hijacked by SARS-CoV-2, highlighting potential therapeutic targets for COVID-19.

## Results

### Multiomics profiling of SARS-CoV-2-infected lung epithelial cells

**Fig. 1:**
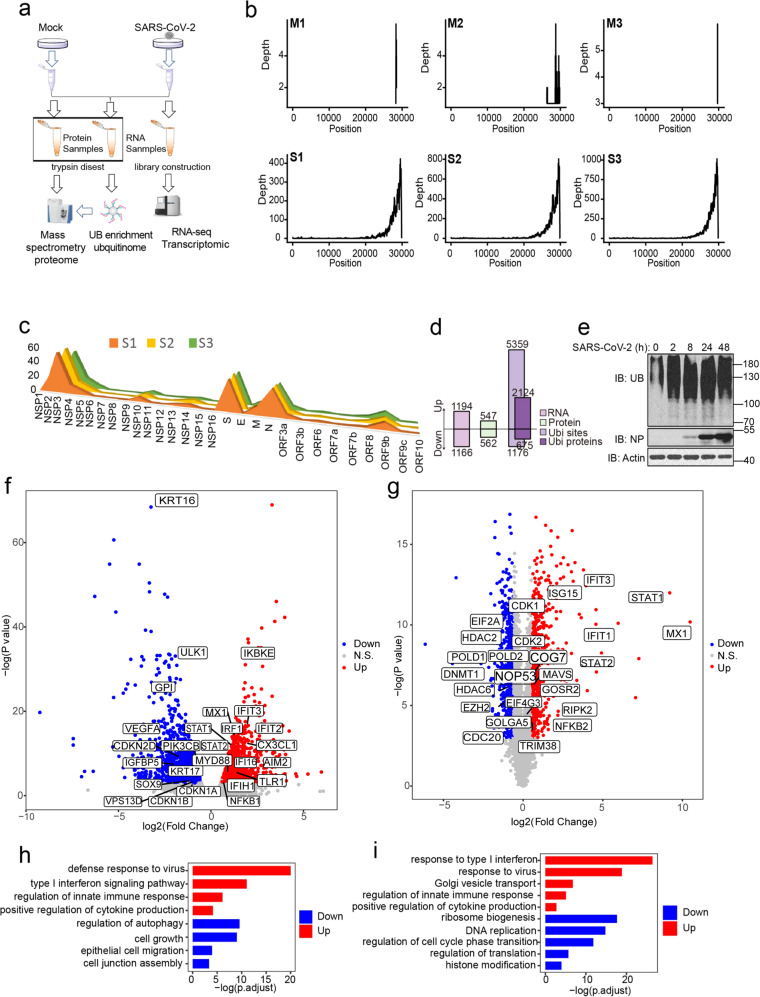
Multiomics profiling of SARS-CoV-2-infected lung epithelial cell. **a** The cartoon outlines the study design. Mock and SARS-CoV-2-infected cells were harvested and lysed with 1% SDS lysis buffer. Aliquots of all samples were analyzed by mass spectrometry (MS) to measure changes in protein abundance upon infection. Another identical samples were enriched for ubiquitinated proteins and then subjected to MS. All conditions were performed in biological triplicate. All ubiquitination sites and protein identifiers were mapped to human and SARS-CoV-2 protein orthologs. The transcriptome data of lung epithelial cells infected with SARS-CoV-2 from our previous study were used for integrated analysis. **b** Genome coverage of viral RNA reads from mock or SARS-CoV-2-infected cells. M1, M2 and M3 marks the three mock samples and S1, S2 and S3 marks the three infection samples. **c** Proteome coverage of viral polypeptide reads from mock or SARS-CoV-2 infected cells. **d** The numbers of distinct transcripts, proteins and ubiquitination sites that are significantly up or downregulated with fold-change greater than 1.5 at 24 h after infection (relative to mock infection, adjust *p*-value < 0.05). **e** Human lung epithelial cells Calu3 were infected with SARS-CoV-2 at MOI of 1 for indicated time. Total ubiquitin conjugates were determined by western blotting using anti-UB and the infection efficiency was determined by western blotting using anti-NP. Actin was used as a loading control. **f** Volcano plot shows the differential gene expression at 24 h after SARS-CoV-2 infection. Cell proliferation and innate immunity-associated genes were marked. **g** Gene Ontology enrichment analysis of the significantly upregulated genes (red) and downregulated genes (blue) after SARS-CoV-2 infection. Selected terms are shown, adjusted *p*-value is indicated. **h** Volcano plot shows the differential protein abundance at 24 h after SARS-CoV-2 infection. DNA replication and innate immunity-associated proteins were marked. **i** Gene Ontology enrichment analysis of the significantly upregulated proteins (red) and downregulated proteins (blue) after SARS-CoV-2 infection. Selected terms are shown, adjusted *p*-value is indicated

To investigate whether host cells deploy different patterns of ubiquitination to cope with SARS-CoV-2 infections, Calu3 cells were harvested in three biological triplicates at 24 h after SARS-CoV-2 infection (S) or mock infection (M) for global ubiquitinomic and proteomic analyses using mass spectrometry (MS) (Fig. 1a and Supplementary Tables 1 and 2). The transcriptome data of lung epithelial cells infected with SARS-CoV-2 from our previous study^17^ were used for integrated analysis (Supplementary Table 3). SARS-CoV-2 protein and RNA were only detected in the infected cells (Fig. 1b, c). Consistent with previous report,^18^ the coverage of the 3′ end of the viral genome was substantially higher than that of the 5′ end, partially due to the sequencing method (Fig. 1b and Supplementary Fig. 1a). MS also showed that the abundance of structural proteins (S, M, and NP) was significantly higher than that of nonstructural proteins (NSP proteins). Moreover, the abundance of proteins encoded by the PP1a open-reading frame (NSP1-NSP11) was higher than those encoded by the PP1b reading frame region (NSP12-NSP16) (Fig. 1c and Supplementary Fig. 1b). This pattern may be an economical and practical strategy for SARS-CoV-2 infection. A large number of structural proteins are required to be packaged into new virus particles, while nonstructural proteins regulate viral replication primarily through their enzyme activity and are less required.

Principal component analysis (PCA) of the samples further revealed a clear separation of mock and infected samples, indicating that the transcriptome, proteome and ubiquitinome of SARS-CoV-2-infected cells were all significantly different from those of non-infected cells (Supplementary Fig. 1c). Genes, proteins and ubiquitination sites with a fold-change less than 1.5 or an adjusted *p*-value greater than 0.05 were filtered out. After quality control, a total of 1194 genes and 547 proteins were upregulated, and 1166 genes and 562 proteins were downregulated (Fig. 1d and Supplementary Tables 1 and 3). By contrast, the ubiquitination of 5359 lysine sites on 2124 proteins were found to be increased and the ubiquitination of 1176 lysine sites on 675 proteins to be decreased. The increased ubiquitination sites detected using MS were far more abundant than the decreased sites (Fig. 1d and Supplementary Table 2). Consistently, immunoblotting also showed an increase in total ubiquitin conjugates post SARS-CoV-2 infection (Fig. 1e). In addition, the protein counts with differential ubiquitination significantly outnumbered the protein counts with differential expressions during SARS-CoV-2-infection, suggesting that alterations in the PTMs of the intracellular proteins are more flexible and efficient than direct changes in the protein level in response to emergency stress such as virus infection.

Unlike previous reports,^5,7,19^ we observed that SARS-CoV-2-infected lung epithelial cells exhibited an increase in not only inflammatory cytokine transcription, but also in IFN-stimulated gene (ISG) expression (Fig. 1f and Supplementary Fig. 1d). This result was also confirmed in Calu3 cells using RT–qPCR (Supplementary Fig. 1e). Gene Ontology (GO) enrichment analysis indicated that several terms related to the innate immune response were upregulated, while the autophagy, cell proliferation and tight junction relevant pathways were downregulated in the SARS-CoV-2-infected cells (Fig. 1g). The proteomic analysis also showed a strong innate immune response activated in SARS-CoV-2-infected Calu3 cells (Fig. 1h and Supplementary Fig. 1f, g). Several terms related to cell cycle and DNA replication were also downregulated at protein levels revealed by the GO analysis (Fig. 1i). These transcriptomic and proteomic responses in vitro are partially consistent with the immune responses of COVID-19 patients, indicating a successful mimicking of natural infection by SARS-CoV-2-infected lung epithelial cells.

### Host innate immune signaling pathways were modified by SARS-CoV-2

**Fig. 2:**
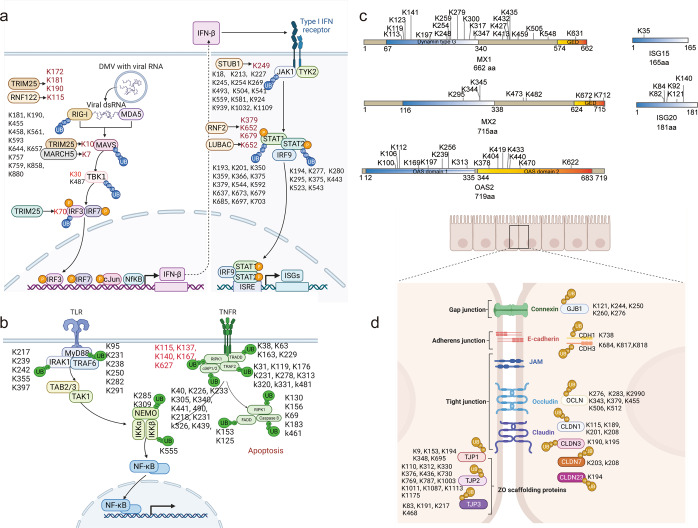
Ubiquitinomic profiling reveals the pathogenic signaling pathways activated by SARS-CoV-2. The Figures were generated from online tools BioRender. **a** Proteins with altered ubiquitination after SARS-CoV-2 infection were enriched in RIG-I-MAVS and JAK-STAT signaling pathway. Sites marked in red were reported, and the corresponding E3 ligases were also marked nearby. **b** The ubiquitination sites on the proteins in TLR and TNF signaling pathway were identified by MS during SARS-CoV-2 infection. The five sites on RIPK1 marked in red have also been identified by mass spectrometry in other study. **c** The diagram shows the ubiquitination sites on multiple ISGs during SARS-CoV-2 infection. **d** The ubiquitination sites on the proteins in tight junctions signaling pathway were identified by MS during SARS-CoV-2 infection

One protein contains multiple lysine sites, and the change of ubiquitination on each site is not consistent, which is actually conducive to more precise regulation of signal transduction. Proteins like XIAP, ZDHHC5, ZDHHC3, and TRAF2 only have sites of increased ubiquitination after SARS-CoV-2 infection, and proteins like ISG15, YWHAZ, and TMEM41B only have decreased ubiquitination sites, while proteins like TRIM25, STAT3, and RIG-I (*DDX58*) contained both increased and decreased ubiquitination sites (Supplementary Fig. 2a). KEGG pathway enrichment analysis was performed on all the proteins with altered ubiquitination. Consistently, the ubiquitinomic data also suggested SARS-CoV-2 infection affected innate immune signaling pathways, including the IFN and inflammation pathways (Fig. 2a, b and Supplementary Fig. 2b–d). Proteins in the RIG-I-MAVS and JAK-STAT signaling pathways were identified to be ubiquitinated, like RIG-I, MAVS, TBK1, JAK1, and STAT1, etc. (Fig. 2a). All the ubiquitination sites (including those with no significant differences) identified by MS on the proteins in the RIG-I-MAVS and JAK-STAT signaling pathways were marked (Fig. 2a). Among the sixteen ubiquitination sites on RIG-I, K172, K181, and K190 were reported to be catalyzed by TRMI25,^13^ and K115 ubiquitination was catalyzed by RNF122,^20^ but the other twelve sites have not been identified (Fig. 2a). The ubiquitination of K7 and K10 on MAVS pathway were reported to be catalyzed by MARCH5^21^ and TRIM25.^22^ The ubiquitination of K30 on TBK1 and K70 on IRF3 were also identified in other studies.^23–25^ However, a newly identified site on TBK1 (K487) has not been reported, leaving the corresponding regulatory roles underexplored. Notably, most of the ubiquitination sites on JAK1, STAT1, and STAT2 were unreported (Fig. 2a), including fifteen ubiquitination sites on JAK1, fifteen sites on STAT1, and eight sites on STAT2. The linear ubiquitination at Lys652 was reported to inhibit STAT1 binding to the type-I interferon receptor IFNAR^26^ and the ubiquitination at Lys249 mediated by STUB1 promoted JAK1 degradation.^27^ Subsequent studies need to clarify whether these newly identified ubiquitination sites are capable of regulating signal transduction and whether they are specific to the SARS-CoV-2 infection process.

Another interesting result observed was that high expression of a series of antiviral ISGs was induced 24 h after SARS-CoV-2 infection (Fig. 1f, h), but the virus still replicated efficiently (Supplementary Fig. 1e). Especially, many ISGs were modified with ubiquitin during viral infections such as MX1, MX2, OAS2, ISG15, and ISG20, which has rarely been reported before (Fig. 2c). Subsequent determination of whether ubiquitination of these ISG is an immune escape strategy used by SARS-CoV-2 is of great importance.

Cytokine storm is a major feature of severe COVID-19,^3^ but the underlying mechanisms are currently unclear. Our ubiquitinomic data suggested that multiple potential pro-inflammatory signaling pathways such as P38/MAPK, PI3K-AKT, EGFR/VEGFR, TLR, and TNF were tampered with by SARS-CoV-2 (Fig. 2b and Supplementary Fig. 2b–d). An amount of previously unreported ubiquitination sites on the proteins in the TLR and TNF signaling pathways were identified, such as MyD88, NEMO, TRADD and TRAF2 and RIPK1 (Fig. 2b). Particularly, the multiple ubiquitination sites on RIPK1 identified using MS are consistent with previous studies.^28^ The ubiquitination of K627 was increased during SARS-CoV-2 infection, which is crucial for RIPK1 activation^28^ (Fig. 2b). RIPK1 was activated during SARS-CoV-2 infection, and inhibition of RIPK1 blocked SARS-CoV-2 infection and inflammatory cytokine production.^17^ We provided novel targets for COVID-19 therapies by determining how the SARS-CoV-2 infection activated these inflammatory signaling pathways.

In addition, SARS-CoV-2 infection also affected the ubiquitination of proteins involved in other physiological activities, like tight junctions. The ubiquitination of several tight junction molecules, such as Connexin, E-cadherin, Occludin and Claudin, has been identified, and ZO-scaffolding proteins that regulate tight junction formation were also ubiquitinated (Fig. 2d). The epithelial barrier function and tight junctions were reported to be disrupted during SARS-CoV-2 infection,^29^ which may be related to the ubiquitination of proteins in tight junction signaling pathways.

Altogether, the present ubiquitinomic showed that SARS-CoV-2 infection potentially activated multiple innate immune signaling pathways in lung epithelial cells, and a number of previously unreported lysine sites were identified. These results not only provided novel targets for COVID-19 therapies, but also provided more information for the study of innate immune signaling pathways.

### SARS-CoV-2 hijacks the ubiquitination of host proteins

**Fig. 3:**
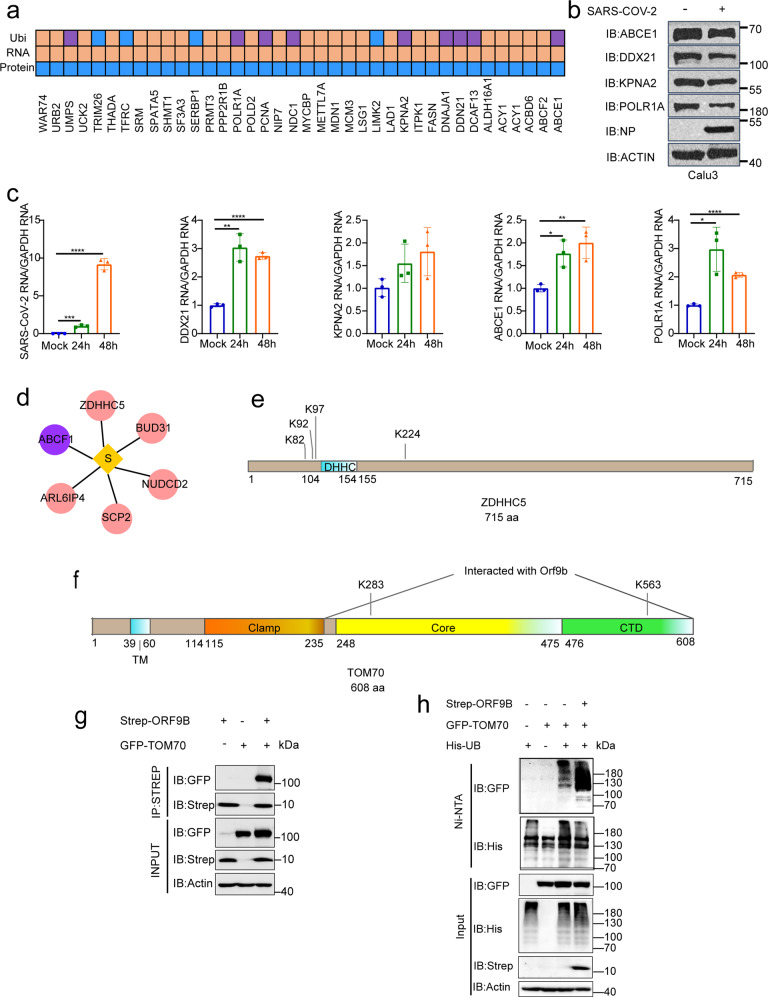
SARS-CoV-2 hijacked host proteins ubiquitination. **a** Heatmaps show the genes whose RNA levels significantly increase but protein levels significantly reduced and the ubiquitination were changed. Purple dots indicate proteins that contain both up-ubiquitinated and down-ubiquitinated sites. Blue dots indicate proteins that only contain down-ubiquitinated sites and orange dots indicate proteins that only contain up-ubiquitinated sites. **b**, **c** Calu3 cells were infected with SARS-CoV-2 at a MOI of 1 for 24 h. The cells were lysed with 2× laemmli sample buffer and analyzed by western blotting using indicated abs (**b**). The indicated RNA was quantified by RT–qPCR (**c**). Paired *t*-test was used in RT–qPCR analysis (*, *p* < 0.05, **, *p* < 0.01, ***, *p* < 0.001 and ****, *p* < 0.0001). **d** The ubiquitination of Spike interacting host proteins was changed after SARS-CoV-2 infection. Purple dots indicate proteins that contain both up-ubiquitinated and down-ubiquitinated sites. Pink dots indicate proteins that only contain up-ubiquitinated sites. **e** The ubiquitination of four lysine sites on ZDHHC5 were all increased during SARS-CoV-2 infection. **f** The ubiquitination of two lysine sites on TOM70 were both increased during SARS-CoV-2 infection. **g** HEK-293T cells were co-transfected with GFP-TOM70 and Strep-ORF9B for 24 h. The cell lysates in 1% NP-40 lysis buffer were analyzed by immunoprecipitation using anti-Strep affinity gel and followed by western blotting using indicated antibodies. **h** HEK-293T cells were co-transfected with GFP-TOM70, HIS-UB and Strep-ORF9B or vector control. The ubiquitination of TOM70 was analyzed by immunoprecipitation using Ni-NTA agarose and followed by western blotting using indicated antibodies

Degradation of host proteins by the ubiquitin–proteasome system was used by viruses to escape host immunity or activate some pathogenic signaling pathways. In vitro treatment with the proteasome inhibitor MG132 inhibited some viral infection,^30,31^ including SARS-CoV-2 (Supplementary Fig. 3a). Of note, we found a total of 38 markers with increased RNA levels but decreased protein levels, to which ubiquitination showed a significant change after SARS-CoV-2 infection (Fig. 3a). Some of them were confirmed by western blotting (WB) and RT–qPCR (Fig. 3b, c). DDX21^32^ and KPNA2^33^ were reported to regulate the host antiviral immunity, and ABCE1^34^ and POLR1A^35^ could regulate cell proliferation. These data indicated that SARS-CoV-2 possibly hijacked the ubiquitin–proteasome system to degrade these proteins, thus weakening the innate immunity and promoting cell proliferation to facilitate viral propagation.

The SARS-CoV-2-host interactome was constructed in several studies through affinity-purification–mass spectrometry (AP-MS) or proximity-dependent biotinylation (BioID).^36–40^ We integrated the interaction data from these studies to construct a relatively complete set of SARS-CoV-2-host interactome (Supplementary Table 4). Interestingly, we found that the ubiquitination of many host proteins that interact with SARS-CoV-2 was also changed after viral infection (Supplementary Fig. 3b), such as ZDHHC5, BUD31, SCP2, ARL6IP4, and NUDCD2, which were reported to interact with the SARS-CoV-2 Spike protein (Fig. 3d). ZDHHC5 is a palmitoyltransferase that interacts with the SARS-CoV-2 Spike protein and mediates its palmitoylation.^41^ Palmitoylation of viral proteins is known to be involved in virus assembly and infection.^42^ Interestingly, the ubiquitination of the four lysine sites on ZDHHC5 was all increased during SARS-CoV-2 infection (Fig. 3e), which may be important for the regulation of ZDHHC5 activity. SARS-CoV-2 ORF9B is known to suppress type I IFN responses by targeting the mitochondrial protein TOM70.^43^ The ubiquitination of K283 and K563 in TOM70 was both increased during SARS-CoV-2 infection (Fig. 3f). We confirmed that ORF9B interacted with TOM70 and further found that ORF9B can increase the ubiquitination of TOM70 (Fig. 3g, h). In addition, other viral-interacting proteins also exhibited altered ubiquitination after viral infection. For example, M interacting protein SMAD2 and SMAD3 are important players in TGF-beta signaling pathway; N interacting protein G3BP1 and G3BP2 are stress granule proteins and N interacting protein DDX1 and DDX3X are RNA helicases (Supplementary Fig. 3c–e).

### Viral proteins were ubiquitinated by host E3 ligases during SARS-CoV-2 infection

**Fig. 4:**
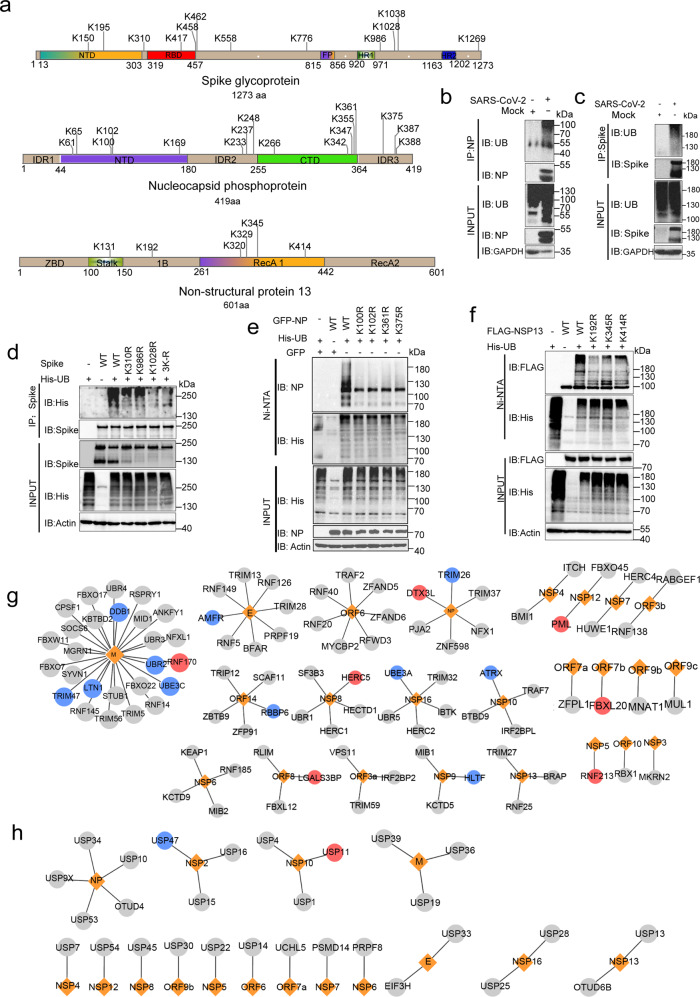
Ubiquitination of SARS-CoV-2 proteins. **a** Mapping of the ubiquitination sites on an alignment of SARS-CoV-2 Spike, NP and NSP13 proteins. **b**, **c** Calu3 cells were infected with SARS-CoV-2 at MOI of 1 for 24 h and cells were lysed with 1% NP-40 lysis buffer. The ubiquitination of NP was analyzed by immunoprecipitation using anti-NP antibody and followed by western blotting using indicated antibodies (**b**). The ubiquitination of Spike was analyzed by immunoprecipitation using anti-Spike antibody and followed by western blotting using indicated antibodies (**c**). **d** HEK-293T cells were co-transfected with mutated or WT Spike and His-UB for 24 h. Cells were lysed with 1% SDS lysis buffer and boiled for 10 min, then diluted with NP-40 buffer for immunoprecipitation using anti-Spike antibody and followed by western blotting using indicated antibodies. 3 R indicates the simultaneous mutation of these three sites (K310R/K986R/K1028R). **e**, **f** HEK-293T cells were co-transfected with mutated or WT NP (**e**), NSP13 (**f**), and His-UB for 24 h. Cells were lysed with 8 μM urea lysis buffer. The ubiquitination of NP (**e**) and NSP13 (**f**) were analyzed by immunoprecipitation using Ni-NTA agarose and followed by western blotting using indicated antibodies. **g**, **h** Base on previous SARS-CoV-2-host interactome, 98 ubiquitin E3 ligases (**g**) and 29 DUBs (**h**) were identified to interacted with SARS-CoV-2 proteins. Viral proteins are shown as orange diamonds, interacting E3 and DUB are shown as circles, protein level increased after virus infection is shown as red and protein level decreased after virus infection is shown as blue. Protein level that did not change significantly after virus infection is shown as gray

In addition to the ubiquitination of a large number of host proteins identified, sixteen SARS-CoV-2 proteins were also identified to be ubiquitinated (Fig. 4a, Supplementary Fig. 4a, and Supplementary Table 5). The largest number of ubiquitination sites was detected on Spike, NP, NSP2 and NSP3 (Fig. 4a and Supplementary Fig. 4a). Most of the ubiquitination sites detected on viral proteins were consistent with the findings in previous study.^6^ We also identified some new sites, such as K150 and K558 on Spike protein and K61, K100, and K387 on NP. The ubiquitination of Spike and NP was further confirmed in SARS-CoV-2-infected Calu3 cells (Fig. 4b, c). To confirm the authenticity of the ubiquitination sites on viral proteins, three lysine sites on Spike, four lysine sites on NP, and three lysine sites on NSP13 were selected to be mutated into arginine. Mutations of these sites inhibited the ubiquitination of Spike, NP, and NSP13, suggesting that these ubiquitination sites are authentic (Fig. 4d–f).

Interestingly, SARS-CoV-2 genome does not encode E3 ubiquitin ligases, indicating that the ubiquitination of viral proteins was catalyzed by host E3 ligases. Based on the interactome data,^36–40^ we found twenty-five viral proteins, which interacted with host E3 ligases (e.g., ORF8 with FBXL12, NSP8 with HERC5, ORF8 with LGALS3BP) and sixteen viral proteins, which interacted with DUB (e.g., NSP2 with USP47, NSP10 with USP11) (Fig. 4g, h). The interaction of ORF8 with FBXL12, NSP8 with HERC5 and ORF8 with LGALS3BP was also confirmed by coimmunoprecipitation (Supplementary Fig. 4b–d).

### The ubiquitination of Spike is critical for viral infection

**Fig. 5:**
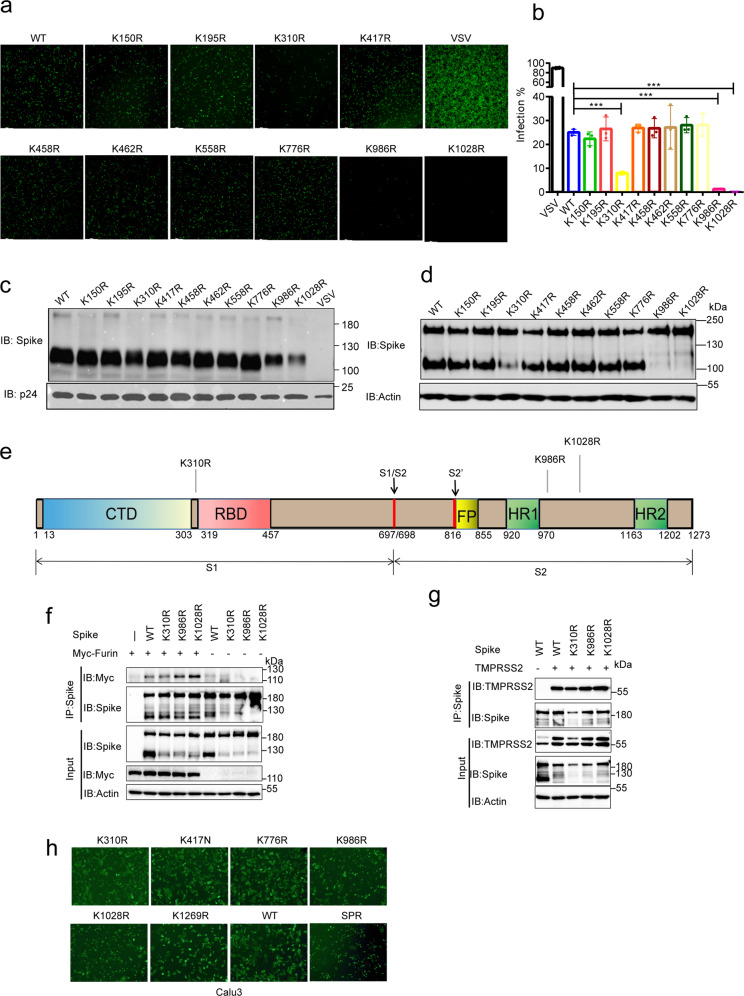
The Ubiquitination of Spike affected the infectivity of SARS-CoV-2. **a**, **b** WT and mutated Spike pseudovirus with GFP were packaged and used to infect 293T-ACE2 cells for 48 h. The image of GFP expression cells was shown (**a**) and the infection efficiency was quantified by calculating GFP-positive cells by Cytation 5 (**b**). **c** WT and mutated Spike pseudovirus were collected for SDS–PAGE, Spike level was determined by western blotting using anti-Spike, and the HIV P24 was used as loading control. **d** WT and mutated Spike plasmids were transfected in HEK-293T cells. Cells were lysed with 2× laemmli sample buffer and analyzed by western blotting using anti-Spike. **e** Localization of the K310, K986 and K1028. **f** WT or mutated Spike plasmid and Myc-Furin plasmid were co-transfected in HEK-293T cells. The cell lysates in 1% NP-40 lysis buffer were analyzed by immunoprecipitation using anti-Spike and followed by western blotting using indicated antibodies. **g** WT or mutated Spike plasmid and TMPRSS2 plasmid were co-transfected in HEK-293T cells. The cell lysates in 1% NP-40 lysis buffer were analyzed by immunoprecipitation using anti-Spike and followed by western blotting using indicated antibodies. **h** HEK-293T cells were co-transfected with WT/mutated Spike and GFP plasmids for 24 h, digested into cell suspension and incubated with Calu3 cells for 2 h. The Syncytia formation was quantified by calculating GFP-positive Calu3 cells. Furin cleavage site deletion mutation was used as positive control (SPR)

Various modifications of Spike protein, such as glycosylation and palmitoylation, affected SARS-CoV-2 infection.^41,44,45^ Although the ubiquitination sites on Spike were identified in a previous study,^6^ whether the ubiquitination of Spike affects SARS-CoV-2 infection remains unclear. We packaged WT and mutant Spike pseudoviruses to investigate the effect of Spike ubiquitination on virus infection. Notably, compared with the WT pseudovirus, mutations of the three sites (K310R, K986R, and K1028R) significantly reduced the infection of SARS-CoV-2 pseudoviruses (Fig. 5a, b and Supplementary Fig. 5a). The level of Spike in the pseudoviruses was detected using WB, and HIV P24 protein was used as the loading control to exclude the effects of mutations on the packaging of pseudoviruses. These three mutations did not affect pseudoviruses packaging, but these three mutations all inhibited the cleavage of Spike (Fig. 5c). For further confirmation, WT and mutant Spike plasmids were transfected into HEK-293T cells and detected using WB. Consistently, The K310R, K986R and K1028R mutations inhibited Spike cleavage (Fig. 5d). Notably, none of the three sites was closed to the furin cleavage region, with K310 located in the S1 subunit, near the RBD region while K986 and K1028 in the S2 subunit (Fig. 5e and Supplementary Fig. 5b). Therefore, we speculated that these 3 mutations would affect the binding of Spike to the two proteases: Furin and TMPRSS2. To confirm that, WT or mutated Spike plasmid were co-transfected with Myc-furin or TMPRSS2 in HEK-293T cells, and the Spike complex was pulled down by immunoprecipitation with an anti-Spike antibody. Myc-Furin and TMPRSS2 were detected in Spike immunoprecipitates, but the K310R, K986R and K1028R mutations did not weaken the binding of Furin and TMPRSS2 (Fig. 5f, g). Neither did these three mutations affect the formation of Spike protein trimers (Supplementary Fig. 5c).

Failure to cleave the Spike protein into S1 and S2 subunits effectively reduced the fusion of the virus to the cell membrane. To detect whether these three mutations affect membrane fusion, HEK-293T cells with GFP and Spike co-expression were digested into a cell suspension and incubated with Calu3 cells for 2 h to determine whether these three mutations affected membrane fusion. Calu3 cells that successfully undergoing membrane fusion expressed GFP. Consistently, these three mutations indeed inhibited syncytia formation (Fig. 5h). This finding could also be replicated in Vero-E6 cells (Supplementary Fig. 5d). Collectively, the SARS-CoV-2 Spike protein was ubiquitinated during virus infection, and the ubiquitination of K310, K986 and K1028 affected the cleavage of Spike, thus affecting SARS-CoV-2 infection.

### Screening of E3/DUB that affect SARS-CoV-2 infection

**Fig. 6:**
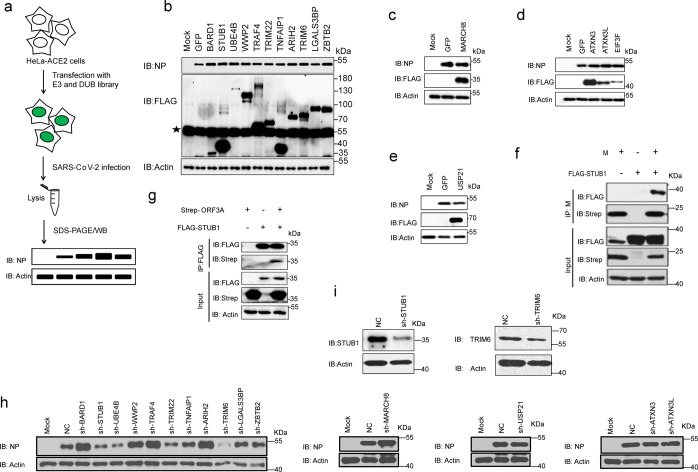
Screening the E3 ligases and DUBs that affect SARS-CoV-2 infection. **a** The cartoon outlines the study design. HeLa-ACE2 cells were transfected with 238 E3 ligases and 39 DUBs library or GFP-vector control for 24 h, and then infected with 0.3 MOI SARS-CoV-2. Cells were harvested at 24 h after infection and lysed with 2× laemmli sample buffer and analyzed by western blotting using anti-NP. Actin was used as the loading control. **b** Eleven E3 ligases were identified to increase SARS-CoV-2 infection. The asterisk represents the non-specific band. **c** E3 ligases MARCH8 was identified to suppress SARS-CoV-2 infection. **d** DUBS ATXN3, ATXN3L and EIF3F have been identified to promote SARS-CoV-2 infection. **e** DUB USP21 was identified to suppress SARS-CoV-2 infection. **f** HEK-293T cells were co-transfected with M and FLAG-STUB1 expression vectors as indicated for 24 h. The cell lysates in 1% NP-40 lysis buffer were analyzed by immunoprecipitation using anti-M antibody and followed by western blotting using indicated antibodies. **g** HEK-293T cells were co-transfected with Strep-ORF3A and FLAG-STUB1 expression vectors as indicated for 24 h. The cell lysates in 1% NP-40 lysis buffer were analyzed by immunoprecipitation using anti-FLAG affinity gel and followed by western blotting using indicated antibodies. **h** shRNA of the 16 top hits were constructed for loss-of-function verification. Three shRNA were designed for each gene, packaged into lentiviruses. HeLa-ACE2 cells were infected with the lentiviruses for 48 h and rapamycin was used to kill those uninfected cells. These stable knockdown cells were infected with 0.3 MOI SARS-CoV-2 for 24 h and lysed with 2× laemmli sample buffer, analyzed by western blotting using anti-NP. Actin was used as the loading control. **i** The knock-down efficiency of TRIM6 and STUB1 shRNA was analyzed by western blotting using anti-TRIM6 or anti-STUB1 antibodies. Actin was used as the loading control

Given that SARS-CoV-2 hijacked the ubiquitin system to degrade host proteins and regulate host innate immunity, and that viral proteins could also be ubiquitinated depending on host E3 ligases, targeting protein ubiquitination was possibly a new therapeutic strategy for COVID-19. Therefore, we screened a library of two-hundred thirty-eight E3 ligases and thirty-nine DUBs for genes that regulate SARS-CoV-2 infection. HeLa-ACE2 cells were cultured in 24-well plates, transfected with 1 μg of E3/DUB or GFP control plasmid, and infected with SARS-CoV-2 at 0.3 MOI. Cells were collected 24 h after infection, the intracellular viral protein NP was detected using WB (Fig. 6a). After three replicates, twelve of E3 ligases and four of DUBs were shown to affect SARS-CoV-2 infection (Fig. 6b–d and Supplementary Fig. 6). The overexpression of eleven E3 ligases (BARD1, STUB1, UBE4B, WWP2, TRAF4, TRIM22, TNFAIP1, ARIH2, TRIM6, LGALS3BP, and ZBTB2) and three DUBs (ATXN3, ATXN3L, and EIF3F) enhanced SARS-CoV-2 infection, while the overexpression of the E3 ligases MARCH8 and DUB USP21 inhibited SARS-CoV-2 infection (Fig. 6b–d). MARCH8 was previously reported to antagonize Spike and other viral glycoproteins,^46^ and STUB1 was reported to interact with SARS-CoV-2 ORF3A,^6^ suggesting that this high-throughput screening results are reliable. STUB1 was also found to interact with M proteins based on the current interactome data (Fig. 4g). The interaction of STUB1 with ORF3A and M protein was confirmed (Fig. 6f, g), indicating that STUB1 regulated SARS-CoV-2 infection by targeting viral proteins.

Owing to the lack of these E3/DUB inhibitors, we designed 3 shRNA for each gene and packaged them into lentiviruses for loss-of-function verification. We further screened out that STUB1, UBE4B, WWP2, TRIM22, and TRIM6 can effectively regulate SARS-CoV-2 infection in HeLa-ACE2 cells. (Fig. 6h and Supplementary Fig. 7a). The knock-down efficiency of TRIM6 and STUB1 has also been verified (Fig. 6i). The top hits were selected to be verified in Calu3 cells and also show that knock-down STUB1, UBE4B, TRIM22, and TRIM6 suppressed SARS-CoV-2 infection (Supplementary Fig. 7b). The mechanisms by which the E3 ligases affected SARS-CoV-2 infection are not known at present, and need future study. Thus, through a library screen we identified four E3s that affect SARS-CoV-2 infection, potentially providing new targets for anti-SARS-CoV-2 therapy.

## Discussion

The inhibition of viral infection and inflammatory cytokine storms are incredibly important for COVID-19 therapy. A deeper understanding of host perturbations induced by SARS-CoV-2, especially the knowledge of the molecular functions of viral proteins and activation mechanisms of host innate immunity by SARS-CoV-2 infection, is paramount for the discovery of therapeutic targets. We used RNA-seq and a mass spectrometry–based approach to study perturbations in the host transcriptome, proteome and ubiquitinome induced by SARS-CoV-2. Different with previous studies, we chose human-derived lung epithelial cell line Calu3 as the infection model to mimic natural infection, as these cells not only effectively support viral infection but also produce a strong innate immune response.

Type I IFN is well known to potentially restrict viral replication^47^ and also inhibit SARS-CoV-2 in dose-dependent manner.^48^ However, we found that SARS-CoV-2-infected lung epithelial cell induces a strong IFN response in vitro but fails to clear the virus. Multiple strategies to antagonize the host innate immune response used by SARS-CoV-2 has been reported.^49–51^ Especially, the ubiquitination and cross-talk between proteins within IFN signaling cascades potentiate its response, and conversely, viruses weaponize the ubiquitination system to antagonize IFN.^52,53^ For example, the K63-linked polyubiquitination of RIG-I mediated by TRIM25 and RNF135 drives the mitochondrial accumulation of RIG-I and interaction with MAVS.^13,54^ Influenza A virus (IAV) nonstructural protein 1 (NS1) targets TRIM25 and RNF135 and blocks their E3 activity.^52^ Hepatitis C virus (HCV) NS3-4A proteolytically cleaves RNF135.^55^ Here, we proposed that SARS-CoV-2 infection antagonized IFN response possibly by ensnaring ubiquitination. Our study identified many new ubiquitination sites on multiple proteins in the RIG-I-MAVS and JAK-STAT signaling pathway, which may be hijacked by SARS-CoV-2 to combat host immunity. Especially, several ISGs such as OAS, MX1, and ISG20, were ubiquitinated during SARS-CoV-2 infection, which is rarely reported before. Future study will determine whether the ubiquitination of ISGs was induced by SARS-CoV-2 to weaken their antiviral abilities, thus providing new targets for SARS-CoV-2 treatment.

Inflammatory cytokine storms are the main contributor to severity of COVID-19, but the underlying mechanism induced by SARS-CoV-2 infection is unclear. Our previous study showed that SARS-CoV-2 infection activated RIPK1 to induce the expression of a variety of inflammatory cytokines, while RIPK1 inhibitors have been shown to inhibit inflammation caused by SARS-CoV-2 infection both in vitro and in vivo.^17^ Here, we also detected the ubiquitination of multi lysine sites on RIPK1 during SARS-CoV-2 infection, of which K627 ubiquitination has been shown to be critical for RIPK1 activation.^28^ Furthermore, the ubiquitination of proteins in the P38/MAPK, EGFR, PI3K-AKT, TLR, and TNF signaling pathways was also identified, indicating that SARS-CoV-2 may activate these signaling pathways to induce inflammation. Indeed, the activated P38/MAPK, EGFR, and PI3K-AKT signaling was also detected in a previous study, which have examined the phosphorylation landscape of SARS-CoV-2 infection.^5^

The functions of posttranslational modifications of SARS-CoV-2 proteins are of great significance but remain largely unknown despite of previous findings.^5,6^ Using the SARS-CoV-2 pseudovirus model, we found that the ubiquitination of K310, K986, and K1028 on the Spike protein is crucial for virus infection. Although the SARS-CoV-2 genome does not directly encode proteins with ubiquitin ligase functions, the host E3/DUBs may ubiquitinate the viral proteins. An analysis of the virus-host interactome has revealed that viral proteins could bind to host E3/DUBs. Through high-throughput screening, four E3 ligases were identified to promote SARS-CoV-2 infection. STUB1 could interact with SARS-CoV-2 M and ORF3A protein, suggesting that STUB1 may regulate viral infection by catalyzing the ubiquitination of these two viral proteins. However, several E3s that interact with viral proteins have not been identified to affect SARS-CoV-2 infection, such as ORF8 interacting protein FBXL12, possibly be related to the overexpression screening system used in the study. A functional verification experiment is further required. In addition, the downstream targets of the other three E3 ligases are unclear. We hope this dataset will be employed to develop additional therapies for COVID-19 in future.

Taken together, our unbiased multiomics datasets have revealed a landscape of ubiquitination-specific processes hijacked by SARS-CoV-2, including not only the IFN and inflammatory processes, but also many new viral and host targets. This study therefore highlights some potential antiviral and anti-inflammatory therapeutics.

## Materials and methods

### Cells and plasmids

HeLa-ACE2 and HEK-293T cells were routinely cultured in Dulbecco’s modified Eagle’s medium (DMEM; Gibco, 11965092). Calu3 cells were cultured in Minimum Essential Medium (MEM; Gibco, 11095080).

Two-hundred thirty-eight E3 ubiquitin ligase and thirty-nine DUB clones (Supplementary Table 6) were obtained from Shenzhen SunV Biotech (Shenzhen, China). Strep II-tagged ORF9b and NSP8 were generously provided by professor Nevan J. Krogan (University of California San Francisco). The His-Ub plasmid was kindly provided by Professor Junying Yuan (Interdisciplinary Research Center on Biology and Chemistry, Shanghai Institute of Organic Chemistry, Chinese Academy of Sciences). Spike and GFP-TOM70 were purchased from Sino Biological (Beijing, China). FLAG-HERC5 was purchased from Shenzhen SunV Biotech.

Mutations of single Lys (K) residues to Arg (R) in Spike, NP and NSP13 were constructed using a MutExpress II mutagenesis kit (Vazyme Biotech, C214-01). All constructs were verified by DNA sequencing.

### SARS-CoV-2 preparation and infection

The SARS-CoV-2 isolate SZTH-003 was sourced from a COVID-19 patient in Shenzhen Third People’s Hospital. Viral stocks were prepared by propagation in Vero-E6 cells and stored at −80 °C. For infection experiments, Calu3 or HeLa-ACE2 cells were incubated with different MOIs SARS-CoV-2 for 1 h at 37 °C. And then the upper media was replaced with DMEM media supplemented with 2% FBS at the corresponding point in time. All work involving live SARS-CoV-2 were performed in the biosafety level-3 (BLS-3) laboratory of Shenzhen Third People’s Hospital.

### Generation of SARS-CoV-2 pseudovirus

HEK-293T cells were cultured in DMEM supplemented with 10% FBS, then transfected with HIV Gag/Pol, HIV rev, plenti-EGFP and the WT or mutated SARS-CoV-2 Spike expression plasmids using Lipofectamine 2000 reagent (Invitrogen, 11668019). The Spike-pseudotyped virus in supernatant was harvested at 48 h post transfection, carefully clarified by centrifugation at 1200 rpm for 10 min at 4 °C and then filtered through a 0.22 μm membrane (Millipore, SLGPR33RB). The virus was frozen at −80 °C.

ACE2-293T or Vero-E6 cells were seeded in 96-well plates and incubated with WT/mutated Spike pseudovirus for 48 h. The infection efficiency was recorded by fluorescence microscopy and positive clones were automatically quantified by Cytation 5.

### Database search

The resulting MS/MS data were processed using MaxQuant search engine (v.1.6.15.0). Tandem mass spectra were searched against the Homo_sapiens_9606_SP_20200509_SARS-CoV-2_NCBI_virus_all_20201214_2nd.fasta (37,151 entries) connected to reverse decoy database. Trypsin/P as cleavage enzyme allow up to 4 missing cleavages. The mass tolerance for precursor ions was controlled at 20 ppm in First search and in Main search, and the mass tolerance for fragment ions was controlled at 20 ppm. Carbamidomethyl on Cys was designated as fixed modification, and ubiquitination on Lys was designated as variable modification. FDR was adjusted to <1%.

### RNA extraction and transcriptomic analysis

TRIzol™ reagent (Invitrogen, 15596018) was used for total RNA extraction from SARS-CoV-2-infected cells. And then reverse transcribed into cDNAs with a High-Capacity cDNA Reverse Transcription Kit (Takara, RR036A).

The sequencing libraries was generated with the RNA-Seq Library Preparation Kit (Nugen; Redwood City, CA). The library size and quality were evaluated by an Agilent Bioanalyzer 2100. Pooled libraries were sequenced with the Illumina NextSEq 550 platform using a 75-cycle kit and single-end read mode.

The sequenced raw reads were mapped to the human genome (GRCh38) using HISAT2. Raw counts were calculated using featureCounts with gene annotation version v94 (gtf version). The DEGs between SARS-CoV-2-infected and mock-infected cells were identified using DESeq257 with an average fold-change in expression >1.5 and adjusted *p*-value < 0.05. Gene enrichment analysis of DEGs was performed by the R package ClusterProfiler. Only biological processes (BPs) were reported in this study.

### Reverse transcription–quantitative polymerase chain reaction (RT–qPCR)

Total RNA was extracted form SARS-CoV-2-infected cells with TRIzol™ Reagent (Invitrogen, 15596026). Then 1 μg RNA was reverse transcribed into cDNAs. qPCR analysis was used to measure the expression levels of the indicated mRNAs with Power SYBR Green PCR Master Mix (Vazyme, Q311-02). The primers used for RT–qPCR has listed in Supplementary Table 7.

### SARS-CoV-2-host interactome

The high-confidence host-SARS-CoV-2 protein interaction networks were curated from five published works^37–41^ (Supplementary Table 4). The interactions with fold-change no more than 2 were excluded as background. The interactome network was generated by Cytoscape software (https://cytoscape.org/).

### Screening the E3 ubiquitin ligase/DUB library

Briefly, 1 × 10^5^ HeLa-ACE2 cells were seeded in 24-well plates and transfected with E3/DUB plasmids or the control vector. One microgram of plasmid was mixed with 3 μg of PEI in 100 μl Opti-MEM (GIBCO, 31985062), incubated for 10 min and then added to the culture. Twenty-four hours post transfection, cells were infected with SARS-CoV-2 at an MOI of 0.3 for 24 h. The cells were harvested for the western blot-based infectivity analysis. SARS-CoV-2 nucleoprotein (Sino Biological, 40588-T62) was used to evaluate infectivity.

### Immunoprecipitation and western blotting

In immunoprecipitation, HEK-293T cells were harvested with 1% NP-40 lysis buffer (50 mM Tris, pH 7.4, 150 mM NaCl, 1% NP-40, 5 mM glycerophosphate, 5 mM NaF) at 4 °C for 30–60 min. The lysates were centrifuged at 12,000 rpm for 10 min at 4 °C, keeping the supernatant and incubating with the indicated antibodies and Protein G Agarose (Thermo, 20397). After the incubation, beads were washed three times with lysis buffer, and then eluted with 2× Laemmli sample buffer.

Boiled cell lysates were separated by SDS–PAGE gels. Then transferred it onto polyvinylidene difluoride (NC) membranes (Millipore, IPFL10100). Blocking with 5% BSA in TBST buffer, then the blot was orderly probed with primary antibodies and the horseradish peroxidase (HRP)-conjugated secondary antibody. Protein bands were detected using SuperSignal™ West Pico PLUS Chemiluminescent Substrate (Thermo Scientific, 34580). The following antibodies were used: anti-His (TransGen Biotech, F1804), anti-FLAG (TransGen Biotech, F1804), anti-Strep tag II (Abbkine, 8C12), anti-GFP (TransGen Biotech, HC101), anti-Mouse IgG (TransGen Biotech, HS201-01), anti-Rabbit IgG (TransGen Biotech, HS101-01) and anti-Actin (TransGen Biotech, HC201).

### His pull down

HEK-293T cells were harvested with 8 M urea lysis buffer (8 M urea, 10 mM Tris-HCl pH 8.0, 500 mM NaCl, 10% glycerol, 0.1% Triton X-100, 10 mM imidazole) on ice for 1 h. The lysate was incubated with Ni-NTA agarose (Invitrogen, 600442) and rotated for 2 h at 4 °C. After the incubation, agarose was washed with lysis buffer containing 20 mM imidazole three times. Proteins were eluted with elution buffer (0.15 M Tris-HCl pH 6.7, 5% SDS, 30% glycerol, 200 mM imidazole, and 0.72 M β-ME). Eluted samples were subjected to western blotting.

### Statistics and data visualization

Principal component analysis (PCA) was performed using the prcomp function in R software. Heatmaps were made by the R package ComplexHeatmap. Interactions among SARS-CoV-2, human proteins and human ubiquitination sites were visualized using Cytoscape software. The volcano plots and bar plots shown in this study were made by the R package ggplot2. The Venn-diagram was plotted using the R package VennDiagram.

## Supplementary information

Supplementary_Materials

Supplementary Tables

## Data Availability

The genomic sequence of SZTH-003 has been uploaded in the Global Initiative of Sharing All Influenza Data (GISAID, EPI_ISL_406594). The RNA-seq data in this paper have been uploaded in the Genome Sequence Archive for human in National Genomics Data Center, Beijing Institute of Genomics, Chinese Academy of Sciences under the accession number HRA000694, which is publicly accessible at https://ngdc.cncb.ac.cn/gsa-human.
