# Supplementary material for: Multiomics approach reveals the ubiquitination-specific processes hijacked by SARS-CoV-2
Source: Signal Transduct Target Ther. 2022 Sep 7;7:312. doi: 10.1038/s41392-022-01156-y (PMC9449932; doi:10.1038/s41392-022-01156-y)
Supplement: Supplementary file 1 — Supplementary_Materials [file 41392_2022_1156_MOESM1_ESM.docx]

Supplementary Materials for

**Multiomics approach reveals the ubiquitination-specific processes hijacked by SARS-CoV-2**

Gang Xu, Yezi Wu, Tongyang Xiao, Furong Qi, Lujie Fan, Shengyuan Zhang, Jian Zhou, Yanhua He, Xiang Gao, Hongxiang Zeng, Yunfei Li, Zheng Zhang

Correspondence to: Z.Z. zhangzheng1975@aliyun.com

**This PDF file includes:**

Materials and Methods

Supplementary Figure 1 to 7

Supplementary Table 7

**Materials and Methods**

**Samples preparation for MS**

Calu3 cells grow in 15cm dish and were infected with 1 MOI SARS-CoV-2 for 24h. SARS-CoV-2-infected and Mock cells were collected, washed with PBS, and then lysed with 1% SDS lysis buffer (50 mM Tris, pH 7.4, 150 mM NaCl, 1% NP-40, 1% SDS, 5 mM β-glycerophosphate, 2.5 mM sodium pyrophosphate, 5 mM NaF, 200 μM Na3VO4, supplemented with protease inhibitor cocktail) on ice for 30 min. The lysates

were sonicated three times on ice using a high intensity ultrasonic processor (Scientz) in lysis buffer. The remaining debris was removed by centrifugation at 12,000 g at 4 °C for 10 min. Finally, the supernatant was collected and the protein concentration was determined with BCA kit (Thermo Fisher, 23225) according to the manufacturer’s instructions.

The protein sample was added with 1 volume of pre-cooled acetone, vortexed to mix, and added with 4 volumes of pre-cooled acetone, precipitated at -20 °C for 2 h. The protein sample was then redissolved in 200 mM TEAB and ultrasonically dispersed. Trypsin was added at 1:50 trypsin-to-protein mass ratio for the first digestion overnight. The sample was reduced with 5 mM dithiothreitol for 60 min at 37 °C and alkylated with 11 mM iodoacetamide for 45 min at room temperature in darkness. Finally, the peptides were desalted by C18 SPE column.

Dissolve the peptide in IP buffer solution (100 mM NaCl, 1 mM EDTA, 50 mM Tris-HCl, 0.5% NP-40, pH 8.0), transfer the supernatant to the ubiquitinated resin that has been washed in advance (antibody resin Product number PTM-1104, from Hangzhou Jingjie Biotechnology Co., Ltd., PTM Bio), placed on a 4 ℃ rotating shaker, gently shake and incubate overnight. After the incubation, the resin was washed with IP buffer solution 4 times and deionized water twice. Finally, 0.1% trifluoroacetic acid eluent was used to elute the resin-bound peptides for three times, then the eluent was collected and vacuumed freeze and drain. After draining, desalination was done following the instructions of C18 ZipTips, desalinated eluent was vacuumed freeze draining again and then use for LC/MS analysis.

**LC–MS/MS**

The tryptic peptides were dissolved in solvent A (0.1% formic acid, 2% acetonitrile in water), directly loaded onto a home-made reversed-phase analytical column (25-cm length, 100 μm i.d.). Peptides were separated with a gradient from 6% to 22% solvent B (0.1% formic acid in acetonitrile) over 43 min, 22% to 30% in 13 min and climbing to 80% in 2 min then holding at 80% for the last 2 min, all at a constant flow rate of 450 nL/min on a nanoElute UHPLC system (Bruker Daltonics).

The peptides were subjected to Capillary source followed by the timsTOF Pro (Bruker Daltonics) mass spectrometry. The electrospray voltage applied was 1.75 kV. Precursors and fragments were analyzed at the TOF detector, with a MS/MS scan range from 100 to 1700 m/z. The timsTOF Pro was operated in parallel accumulation serial fragmentation (PASEF) mode. Precursors with charge states 0 to 5 were selected for fragmentation, and 10 PASEF-MS/MS scans were acquired per cycle. The dynamic exclusion was set to 24 s.

**MS data analysis**

The raw LC-MS datasets were first searched against database and converted into matrices containing reporter intensity of peptides across samples. The relative quantitative value of each modified peptide was then calculated based on this intensity information by the following steps:

Firstly, the intensities of modified peptides (I) were centralized and transformed into relative quantitative values (U) of modified peptides in each sample. The formula is listed as follow: i denotes the sample and j denotes the modified peptide.

Rij = Iij / Mean(Ij)

If both Proteomics and Post-translational modification profiling were conducted on the same cohort, the relative quantitative value of the modified peptide is usually divided by the relative quantitative value of corresponding protein to remove the influence from protein expression of modifications (note: by checking whether there are protein relative expressions in 2-basic_analysis /MS_identified_information.xlsx to determine if normalization is done).

Firstly, the samples to be compared were selected in pairwise, and the fold change (FC) was calculated as the ratio of the mean intensity for each modification site in two sample groups. For example, to calculate the fold change between sample A and sample B, the formula is listed as following: R denotes the relative quantitative value of the modification site, i denotes the sample and k denotes the modification site.

FCA/B,k = Mean(Rik, i∈A) / Mean(Rik, i∈B)

To calculate the significance of the difference between groups, student's T test was performed on the relative quantitative value of each modification site in the two sample groups, and the corresponding P value was calculated as the significance index. #P value < 0.05# was considered as significant. To make the data conform a normal distribution, the relative quantitative value of modification site was log2 transformed. The formula is listed as following:

Pik = T.test(Log2(Rik, i∈A), Log2(Rik, i∈B))

**Supplementary Figure**


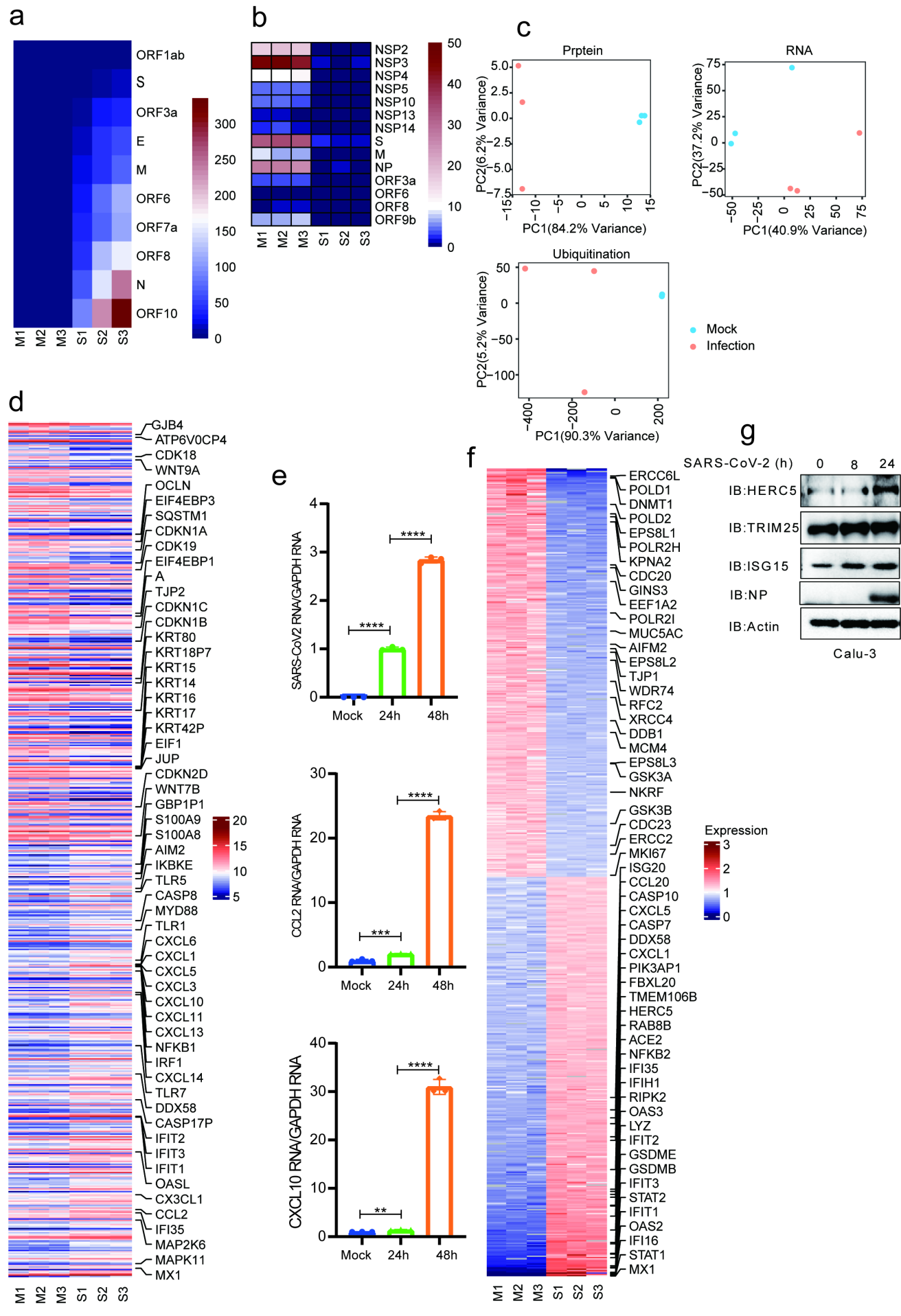


**Figure S1. RNA-seq and Proteomics Data related to Figure. 1**

**a** Heatmaps show the encoded RNA abundance corresponding to the viral proteins in SARS-CoV-2 infected cells.

**b** Heatmaps show the different viral protein abundance in SARS-CoV-2 infected Calu3 cells.

**c** Principal component analysis of transcriptome replicates, proteome replicates and ubiquitinome replicates of mock or SARS-CoV-2 infected cells.

**d** Heatmaps show the differential expression genes (DEGs) between SARS-CoV-2 and mock infected cells. Genes associated with innate immunity and cell proliferation are selectively labeled. (fold change > 1.5, adjusted *p* < 0.05).

**e** Calu3 cells were infected with SARS-CoV-2 at a MOI of 1 for 24h. Total RNA was extracted and reverse transcripted. The levels of indicated proinflammatory cytokines were measured by RT-qPCR. Paired t-test was used in RT-qPCR analysis (**, *p* < 0.01, ***, *p* < 0.001 ****, *p* <0.0001.).

**f** Heatmaps show the difference proteins between mock and SARS-CoV-2 infected Calu3 cells. Genes associated with IFN response, DNA replication and translation are selectively labeled. (fold change > 1.5, adjusted *p* < 0.05).

**g** Calu3 cells were infected with SARS-CoV-2 at a MOI of 1 for 24h. The cells were lysed with 2X laemmli sample buffer and analyzed by western blotting using indicated abs.


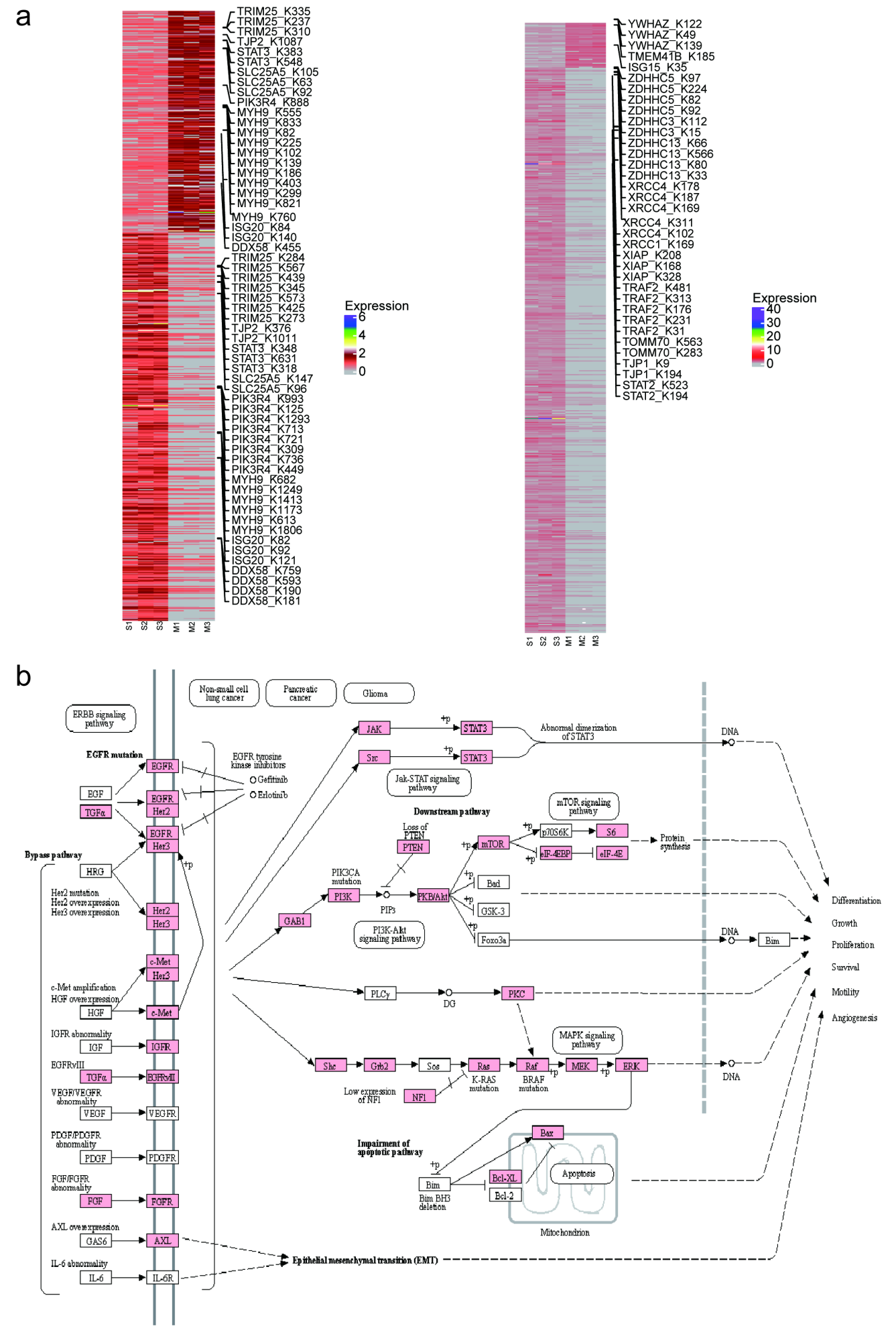

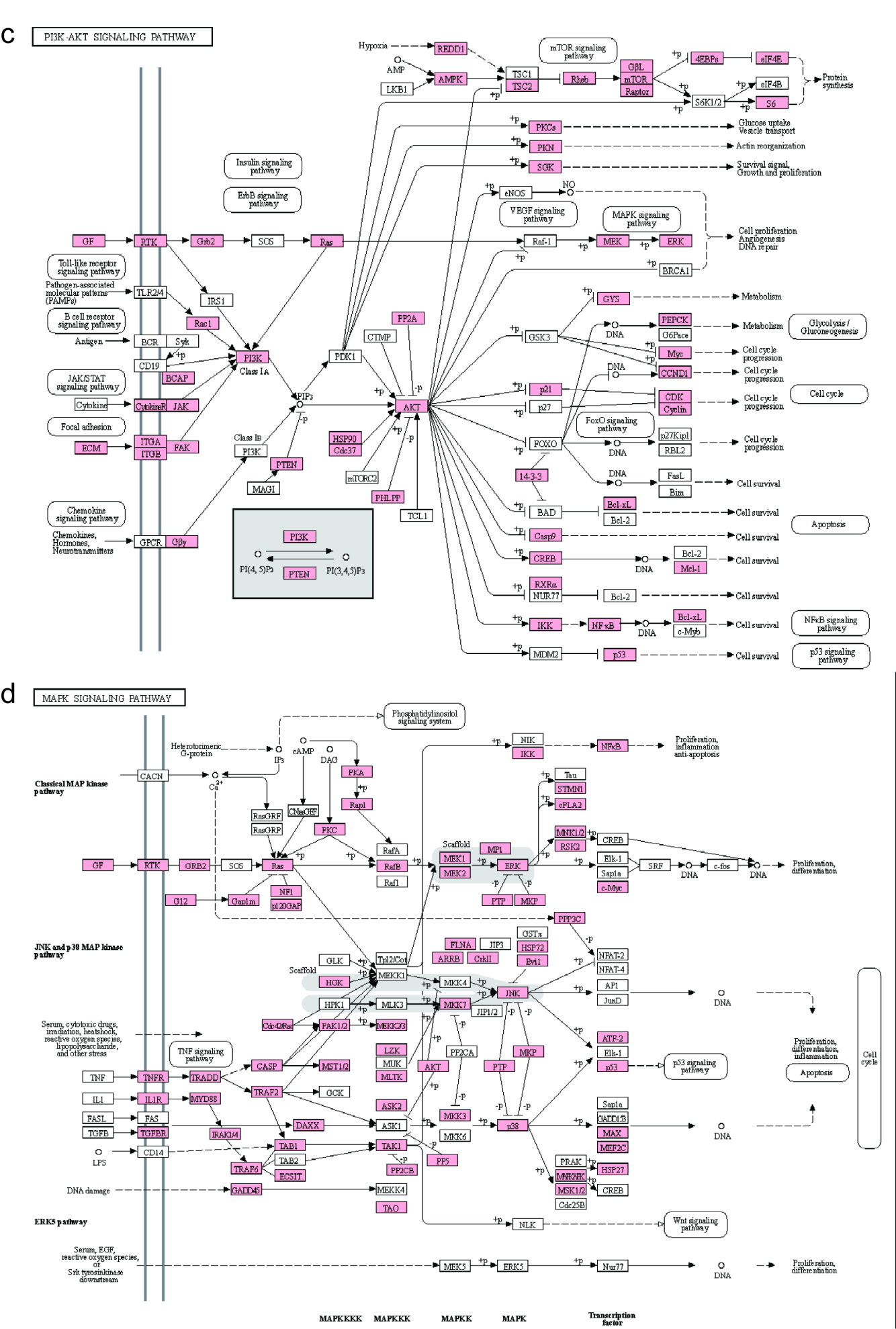


**Figure S2. KEGG signaling pathway enrichment analysis of the proteins with altered ubiquitination, Data related to Figure. 2**

**a** Heatmaps show the difference ubiquitin modification sites between mock and SARS-CoV-2 infected Calu3 cells. The ubiquitination of proteins only increased, only decreased (left) and containing both (right) are selectively labeled. (fold change > 1.5, adjusted *p* < 0.05).

**b** Proteins with altered ubiquitination after SARS-CoV-2 infection were enriched in EGFR signaling pathway.

**c** Proteins with altered ubiquitination after SARS-CoV-2 infection were enriched in PI3K-AKT signaling pathway.

**d** Proteins with altered ubiquitination after SARS-CoV-2 infection were enriched in MAPK signaling pathway.

**
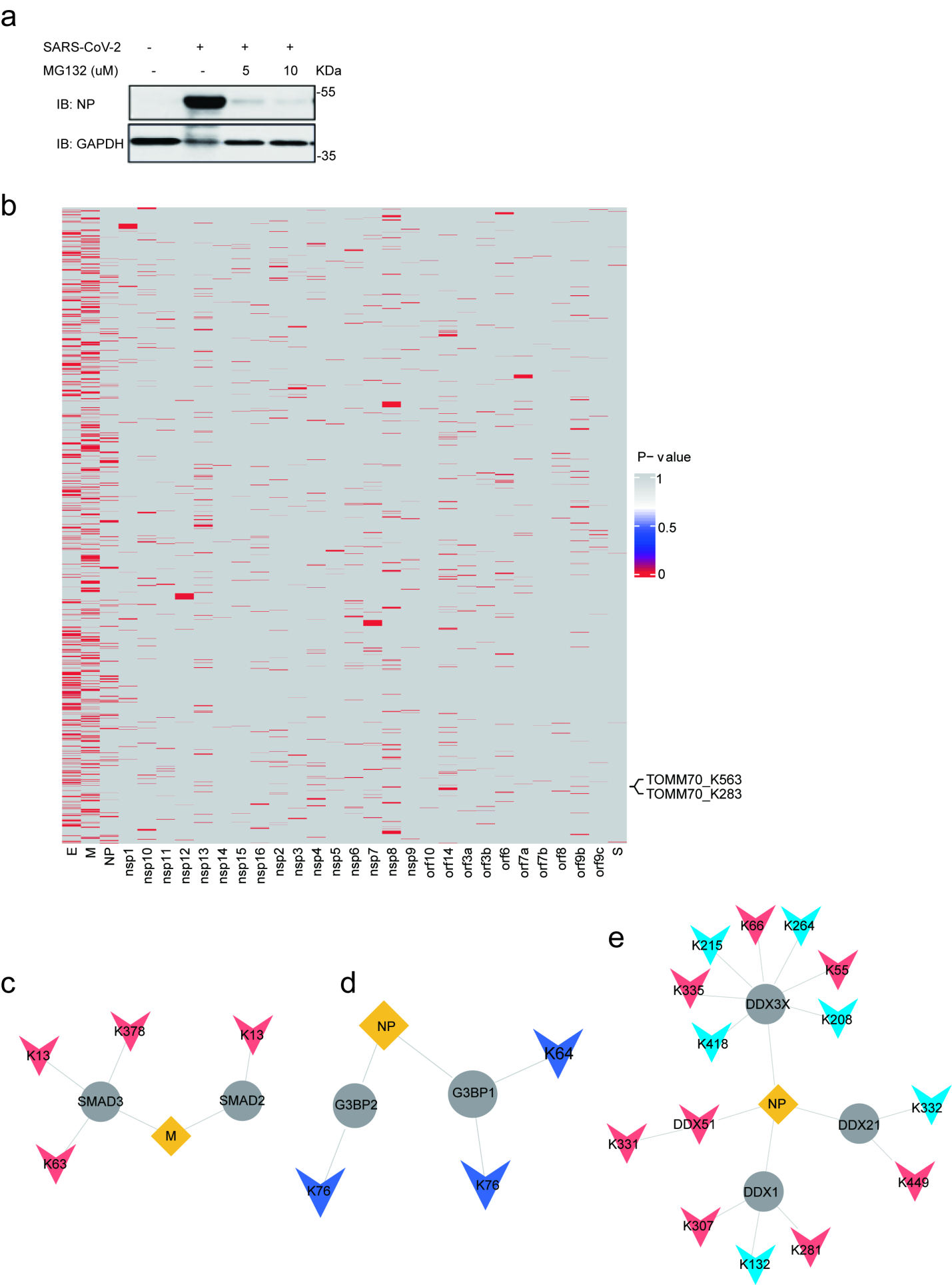
**

**Figure S3. Host proteins ubiquitination were hijacked by SARS-CoV-2，Data related to Figure. 3**

**a** Vero-E6 cells were first infected with SARS-CoV-2 at MOI of 0.3 for 2 h, washed with PBS and replaced with fresh media with 5 and 10μM MG132 or DMSO and incubated for 24h. Viral protein was quantified by western blotting for NP.

**b** Heatmaps show the dynamic changes of the ubiquitination of SARS-CoV-2 interacting host proteins.

**c-e** The ubiquitination of M interacting proteins SMAD3 and SMAD2 in TGF signal pathway was increased after SARS-CoV-2 infection(**c**). The ubiquitination of stress granule proteins G3BP1 and G3BP2 interacting with NP was decreased after SARS-CoV-2 infection (**d**). The changes in ubiquitin modification of RNA helicase DDX family proteins that interact with SARS-CoV-2 NP protein(**e**). Pink dots indicate ubiquitination sites that are upregulated in SARS-CoV-2 infected Calu3 cells. Blue dots indicate ubiquitination sites that are downregulated in SARS-CoV-2 infected Calu3 cells. Grey dots mean ubiquitinated proteins.


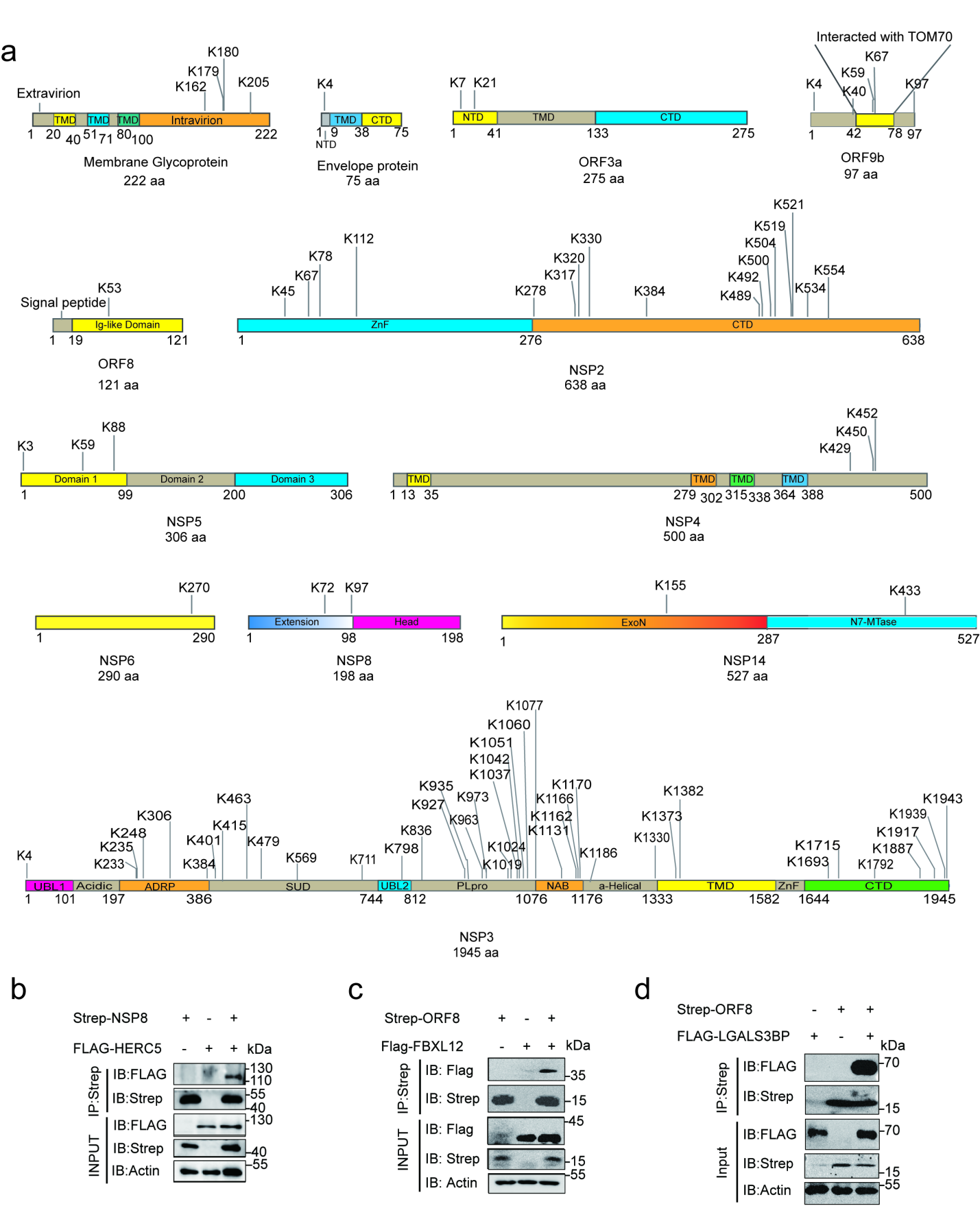


**Figure S4. Viral protein ubiquitination, Data related to Figure. 4**

**a** Mapping of the ubiquitination sites on an alignment of SARS-CoV-2 proteins.

**b** HEK-293T cells were co-transfected with Strep-NSP8 and Flag-HERC5 expression vectors as indicated for 24 h. The cell lysates in 1% NP-40 lysis buffer were analyzed by immunoprecipitation using anti-Strep affinity gel and followed by western blotting using indicated antibodies.

**c** HEK-293T cells were co-transfected with Strep-ORF8 and Flag-FBXL12 expression vectors as indicated for 24 h. The cell lysates in 1% NP-40 lysis buffer were analyzed by immunoprecipitation using anti-Strep affinity gel and followed by western blotting using indicated antibodies.

**d** HEK-293T cells were co-transfected with Strep-ORF8 and FLAG-LGAGS3BP expression vectors as indicated for 24 h. The cell lysates in 1% NP40 lysis buffer were analyzed by immunoprecipitation using anti-Strep affinity gel and followed by western blotting using indicated antibodies.

**
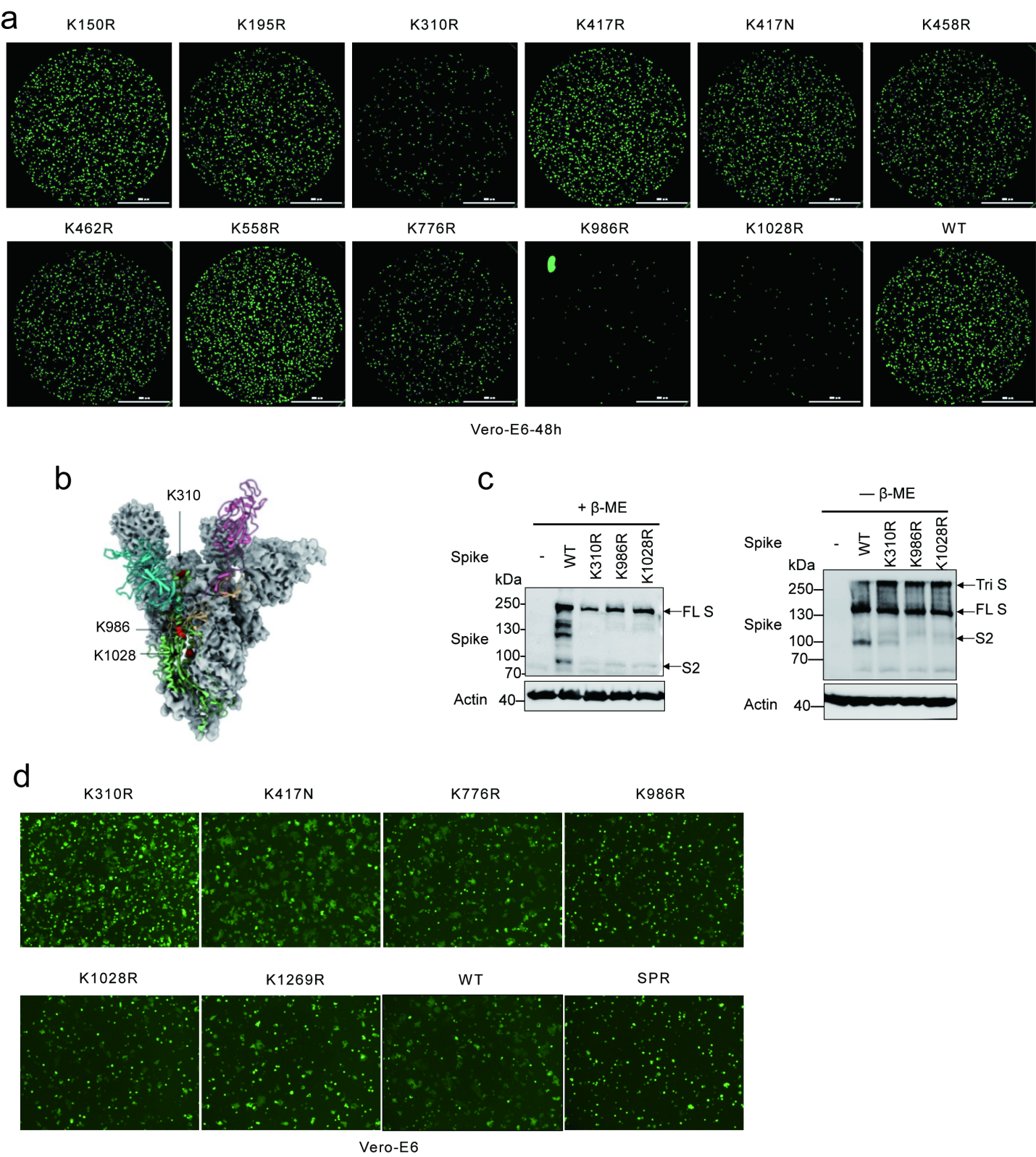
**

**Figure S5. The effect of Spike ubiquitination on SARS-CoV-2 infection, Data related to Figure. 5**

**a** WT and mutated Spike pseudovirus with GFP were packaged and used to infect HEK- Vero-E6 cells for 48h. The infection efficiency was quantified by calculating GFP-positive cells by Cytation 5.

**b** The three ubiquitination sites (310K, 986K and 1028K) were mapped on the structure of SPIKE (PDB ID: 7cwu).

**c** HEK-293T cells were transfected with WT or mutated Spike (K310R, K986R and K1028R) for 24h. The cells were collected and divided into 2 portions, one portion was lysed with 2X laemmli buffer containing β-Mercaptoethanol and the other with lysate without β-Mercaptoethanol, analyzed by western blotting using indicated abs.

**d** HEK-293T cells were co-transfected with WT/mutated Spike and GFP plasmids for 24h, digested into cell suspension and incubated with Vero-E6 cells for 2h. The Syncytia formation was quantified by calculating GFP-positive Vero-E6 cells. Furin cleavage site deletion mutation was used as positive control (SPR).


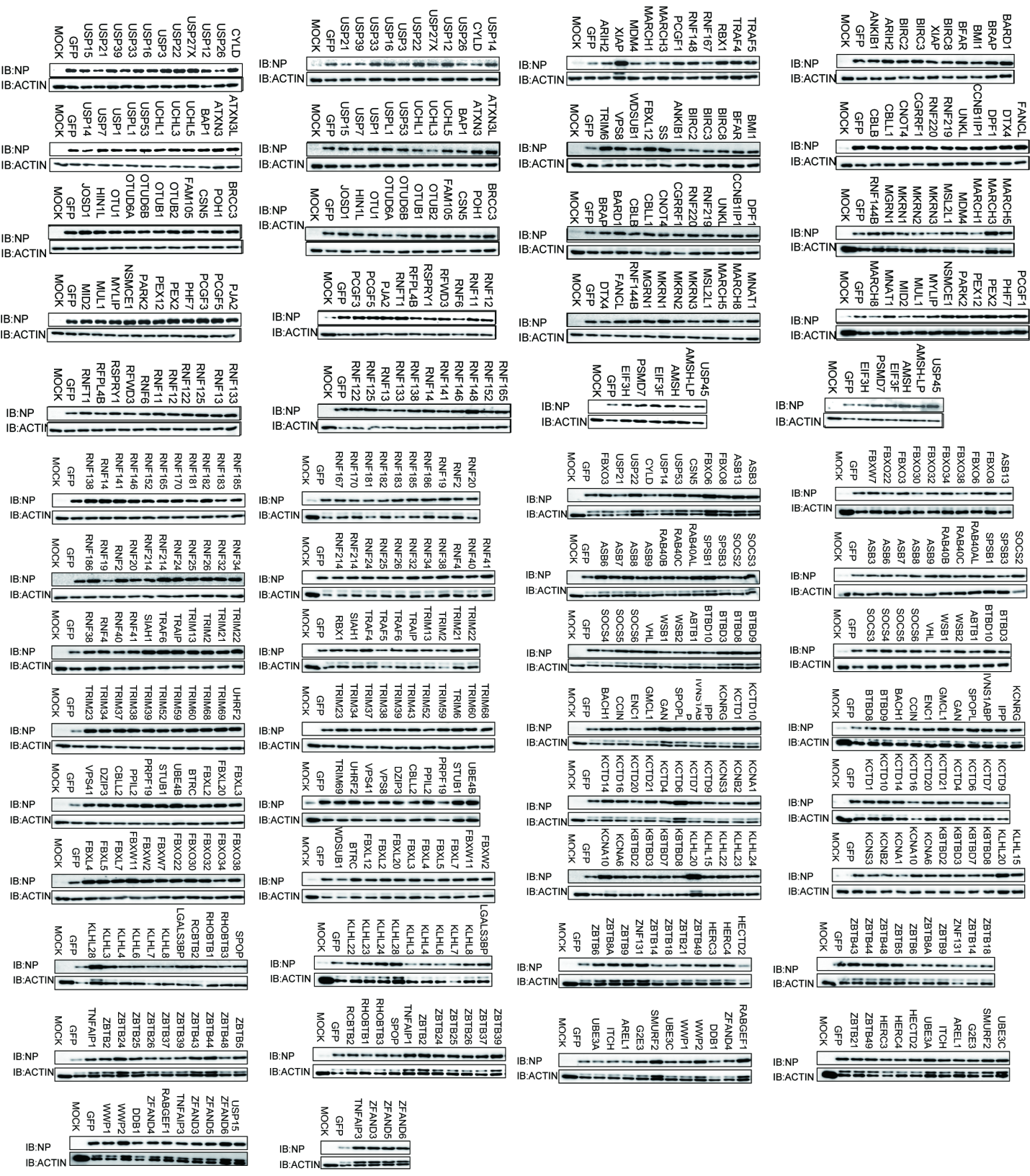


**Figure S6. Screening of E3 ligases and DUBS that affect SARS-CoV-2 infection, Data related to Figure. 6**

238 E3 ligases and 39 DUBs or vector control with GFP label were transfected into HeLa-ACE2 cells for 24h. After confirming that 80 to 90 percent of the cells were GFP positive, cells were transferred to the P3 laboratory to infect with 0.3 MOI SARS-CoV-2 for 24h. Cells were harvested and lysed with 2× laemmli sample buffer and analyzed by western blotting using anti-NP. Actin was used as a loading control.


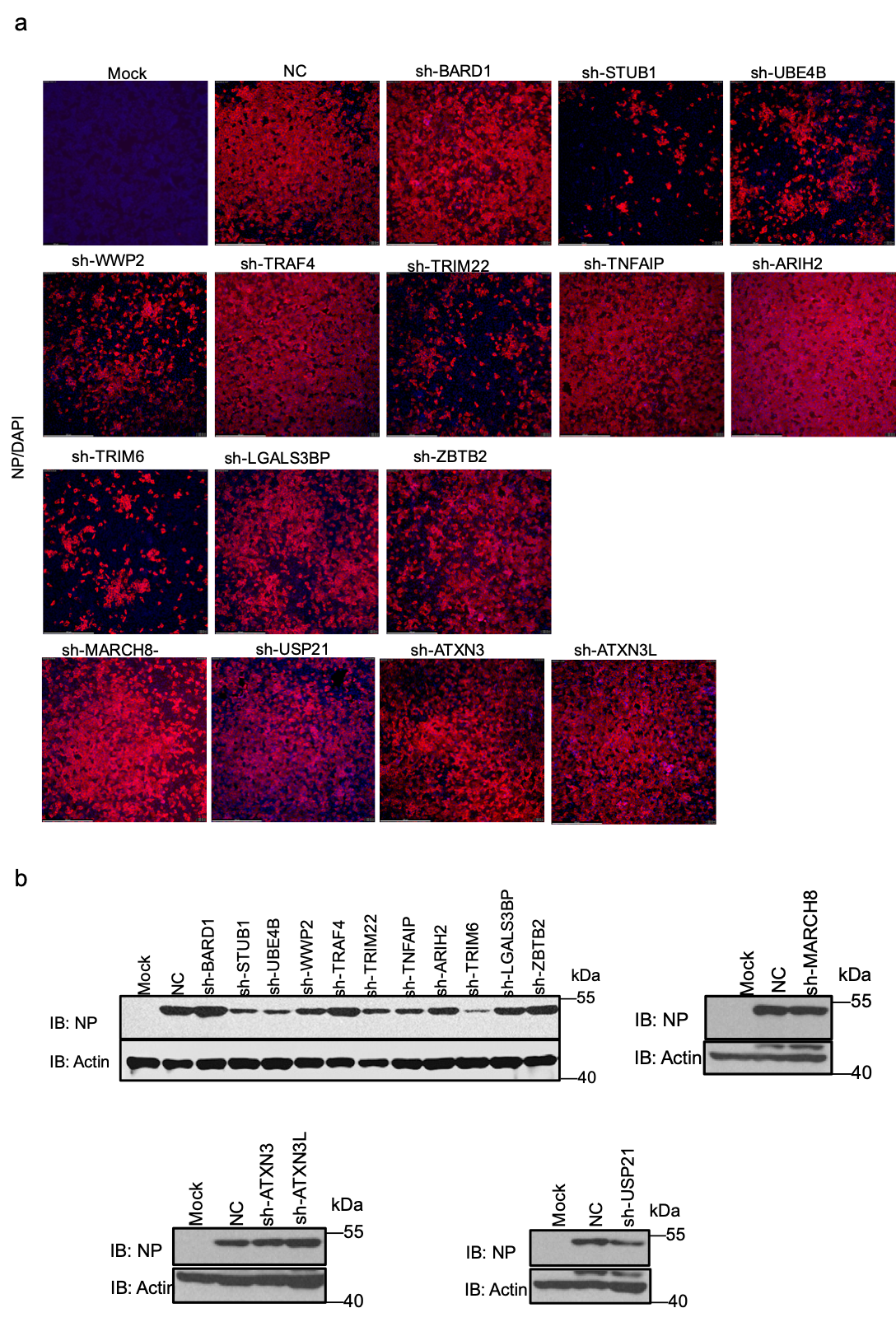


**Fig S7 Validation of the top hits regulating SARS-CoV-2 infection.**

**a** Immunofluorescence assay was used to confirm the 12 E3 and 4 DUBs. Hela-ACE2 cells with stable expression the shRNA of 16 E3s were infected with SARS-CoV-2 for 24h. Cells were collected for WB detection (Fig. 6h). The collected supernatant was centrifuged to remove cell debris and then used to infect Vero-E6 cells for 24h. Cells were fixed with 4% PFA for immunofluorescence analysis. Red is the signal for NP, and blue is the DAPI-stained nucleus signal.

**b** shRNA of the 16 top hits were constructed for loss-of-function verification. Three shRNA were designed for each gene, packaged into lentiviruses. Calu3 cells were infected with the lentiviruses for 48h and rapamycin was used to kill those uninfected cells. These stable knockdown cells were infected with 0.3 MOI SARS-CoV-2 for 24h and lysed with 2× laemmli sample buffer, analyzed by western blotting using anti-NP. Actin was used as a loading control.

**Supplementary Table 7. The list of primers used in this study**
